# A randomized placebo-controlled trial of nicotinamide riboside and pterostilbene supplementation in experimental muscle injury in elderly individuals

**DOI:** 10.1172/jci.insight.158314

**Published:** 2022-10-10

**Authors:** Jonas Brorson Jensen, Ole L. Dollerup, Andreas B. Møller, Tine B. Billeskov, Emilie Dalbram, Sabina Chubanava, Mads V. Damgaard, Ryan W. Dellinger, Kajetan Trošt, Thomas Moritz, Steffen Ringgaard, Niels Møller, Jonas T. Treebak, Jean Farup, Niels Jessen

**Affiliations:** 1 Department of Biomedicine and; 2 Research Laboratory for Biochemical Pathology, Department of Biomedicine, Aarhus University, Aarhus, Denmark.; 3 Steno Diabetes Center Aarhus and; 4 Diabetes and Hormonal Diseases, Aarhus University Hospital, Aarhus, Denmark.; 5 Novo Nordisk Foundation Center for Basic Metabolic Research (CBMR), University of Copenhagen, Copenhagen, Denmark.; 6 Elysium Health, New York, New York, USA.; 7 Magnetic Resonance Research Centre, Department of Clinical Medicine, and; 8 Medical Research Laboratory, Department of Clinical Medicine, Aarhus University, Aarhus, Denmark.

**Keywords:** Aging, Muscle Biology, Human stem cells, Skeletal muscle

## Abstract

**BACKGROUND:**

During aging, there is a functional decline in the pool of muscle stem cells (MuSCs) that influences the functional and regenerative capacity of skeletal muscle. Preclinical evidence has suggested that nicotinamide riboside (NR) and pterostilbene (PT) can improve muscle regeneration, e.g., by increasing MuSC function. The objective of this study was to investigate if supplementation with NR and PT (NRPT) promotes skeletal muscle regeneration after muscle injury in elderly individuals by improved recruitment of MuSCs.

**METHODS:**

Thirty-two elderly individuals (55–80 years of age) were randomized to daily supplementation with either NRPT (1,000 mg NR and 200 mg PT) or matched placebo. Two weeks after initiation of supplementation, skeletal muscle injury was induced by electrically induced eccentric muscle work. Skeletal muscle biopsies were obtained before, 2 hours after, and 2, 8, and 30 days after injury.

**RESULTS:**

A substantial skeletal muscle injury was induced by the protocol and associated with release of myoglobin and creatine kinase, muscle soreness, tissue edema, and a decrease in muscle strength. MuSC content, proliferation, and cell size revealed a large demand for recruitment after injury, but this was not affected by NRPT. Furthermore, histological analyses of muscle fiber area, central nuclei, and embryonic myosin heavy chain showed no NRPT supplementation effect.

**CONCLUSION:**

Daily supplementation with 1,000 mg NR and 200 mg PT is safe but does not improve recruitment of the MuSC pool or other measures of muscle recovery in response to injury or subsequent regeneration in elderly individuals.

**TRIAL REGISTRATION:**

ClinicalTrials.gov NCT03754842.

**FUNDING:**

Novo Nordisk Foundation (NNF17OC0027242) and Novo Nordisk Foundation CBMR.

## Introduction

Skeletal muscle mass is positively correlated with longevity and inversely correlated with negative health outcomes in elderly individuals (1, 2). A sufficient pool of muscle stem cells (MuSCs) is required to repair and maintain skeletal muscle (3). MuSCs mostly reside in a quiescent state, but they are activated and proliferate in response to environmental signals, such as injury. The majority of proliferating MuSCs undergo differentiation and fusion into myofibers, while a minor subset returns to quiescence and thereby maintains the MuSC pool. However, in rodent models of aging, MuSC content and function decline over the course of aging; this is directly related to impaired myofiber regeneration (3).

Nicotinamide adenine dinucleotide (NAD^+^) homeostasis is critical for cell and organismal function. NAD^+^ and associated metabolites act as coenzymes in redox reactions and cosubstrates for other classes of enzymes, including sirtuins and poly(adenosine diphosphate–ribose) polymerases (4). In humans, age and NAD^+^ levels are negatively correlated in several tissues (5–7). Thus, a decline in cellular NAD^+^ levels is a proposed hallmark of aging. NAD^+^ synthesis is largely dependent on the salvage pathway in which nicotinamide phosphoribosyltransferase (NAMPT) is rate limiting (8). Studies have revealed an age-associated decrease in NAMPT levels in certain tissues, including skeletal muscle (9–11). This indicates that lower NAMPT levels are partly responsible for decreased NAD^+^ levels. In murine skeletal muscle, ablation of NAMPT causes substantial NAD^+^-lowering and progressive muscle degeneration (12–14). In these models, muscle function is partly rescued by nicotinamide riboside (NR), which bypasses NAMPT in generation of NAD^+^.

The transition of MuSCs from a quiescent state to cell-cycle entry requires a shift in substrate metabolism, which is partly sirtuin dependent (15). Thus, preclinical studies have shown beneficial effects of NAD^+^-increasing strategies in relation to MuSC quantity, function, and overall muscle regeneration (16, 17). Specifically, NR supplementation was shown to enhance regeneration after muscle injury in aged mice by SIRT1-mediated upregulating of genes related to oxidative phosphorylation in MuSCs (17). In addition, preclinical data suggest that muscle repair can be improved by addition of the polyphenol pterostilbene (PT), a suggested activator of the sirtuin family (18). PT is an analog to resveratrol but with higher bioavailability due to the presence of 2 methoxy groups (19). Investigations of combined NR and PT (NRPT) effects in humans are limited, but recent studies show beneficial effects of NRPT supplementation on functional strength parameters in both elderly individuals and patients with amyotrophic lateral sclerosis (20, 21).

In this study, we investigated whether NRPT supplementation improves recruitment of the MuSC pool and, thereby, skeletal muscle regeneration, after an experimentally induced injury in elderly individuals in a randomized placebo-controlled design.

## Results

### Anthropometrics and activity level.

**Figure 1:**
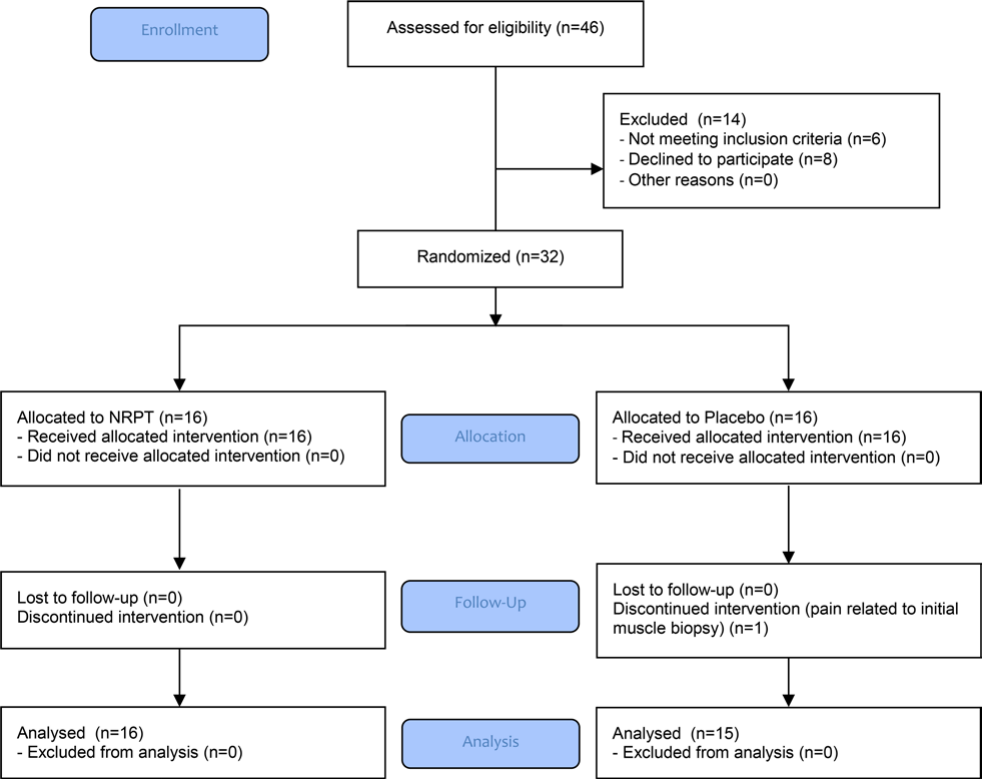
CONSORT flow chart.

**Figure 2:**
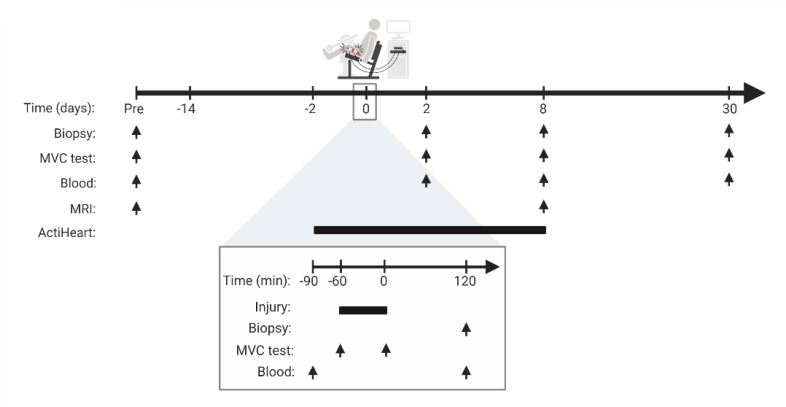
Study overview. Participants were examined before initiation of NRPT or matched placebo supplementation (Pre). Supplementation started 14 days before induction of skeletal muscle injury and continued until 30 days after injury. Skeletal muscle injury was induced at time point 0 by electrically induced contractions combined with eccentric work in a dynamometer (200 repetitions, 100 slow and 100 fast). The bottom line gives a detailed description of the injury day. The arrows indicate timing of skeletal muscle biopsy, MVC test, blood samples, and MR imaging, respectively. Bars indicate time span. Skeletal muscle biopsies were obtained from the uninjured leg prior to starting supplementation and from the injured leg after induction of injury. MVC, maximal voluntary contraction.

Enrollment and data collection were performed from February 2019 to September 2020. Forty-six people were invited for screening, of which 14 did not meet inclusion criteria (*n* = 6) or declined to participate (*n* = 8). Thirty-two participants were enrolled and randomly assigned to supplementation with either NRPT (NR, 1,000 mg/d; PT, 200 mg/d) or matched placebo. A flow chart and detailed study overview are provided in Figure 1 and Figure 2. Thirty-one participants completed the study, with 1 dropout (placebo group) due to unacceptable pain related to the initial muscle biopsy. Anthropometric, activity, and injury protocol data are presented in Table 1.

### Tolerability and compliance.

**Figure 3:**
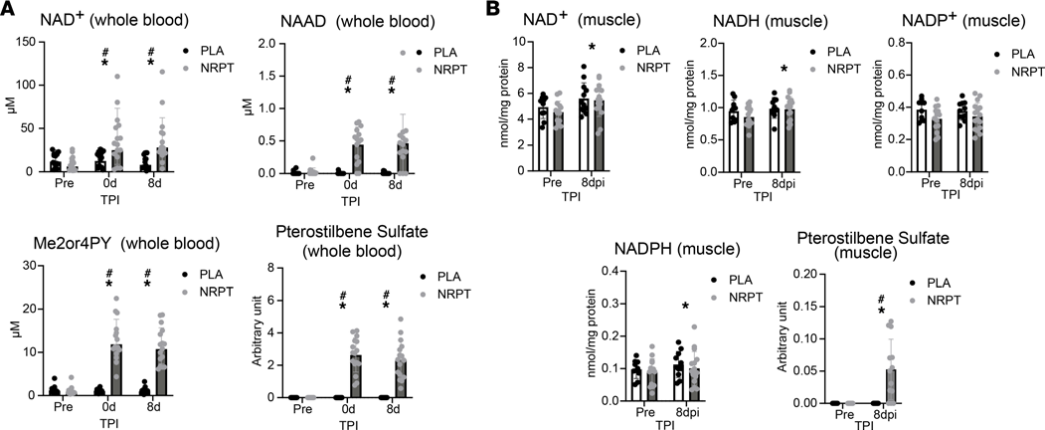
NAD^+^ metabolites and pterostilbene sulfate in whole blood and skeletal muscle. (**A**) Analysis of whole blood revealed an increase in NAD^+^, nicotinic acid adenine dinucleotide (NAAD), N1-methyl-2-pyridone-5-carboxamide/N1-Methyl-4-pyridone-3-carboxamide (Me2/4PY), and pterostilbene sulfate following NRPT supplementation. (**B**) NAD^+^, NADH, and NADPH were increased in skeletal muscle after injury, whereas NADP^+^ was not. NRPT supplementation was able to increase pterostilbene sulfate in skeletal muscle tissue. Data are expressed as mean ± SD and were compared using repeated-measurement mixed-model analysis. For pterostilbene sulfate, each group was tested separately using Friedman’s test, and for supplementation effect, groups were compared at each time point after injury with the Mann-Whitney test. Whole blood: NRPT, *n* = 16; PLA, *n* = 15. Muscle NAD^+^ metabolites: NRPT, *n* = 16; PLA, *n* = 14. Muscle pterostilbene sulfate: NRPT, *n* = 15; PLA, *n* = 11. ^#^*P* < 0.05 between groups; *P < 0.05 vs. Pre. TPI, time after injury.

In general NRPT supplementation was well tolerated. Two individuals reported constipation in the placebo group, whereas transient reflux (*n* = 1) and transient loose stools (*n* = 1) were reported in the NRPT group. The severity was mild in every case. In addition to the low frequency of adverse reactions, compliance rates (measured by tablet counting at the end of the study) were 95.7% ± 4.9% and 95.8% ± 4.0% in the placebo group and the NRPT group, respectively. Blood biochemistry showed no difference between groups after 14 days of supplementation (Supplemental Table 1; supplemental material available online with this article; https://doi.org/10.1172/jci.insight.158314DS1). NRPT supplementation significantly increased whole blood levels of NAD^+^, NAAD, and Me2/4PY as well as pterostilbene sulfate, confirming compliance and uptake of the supplement (Figure 3A). In skeletal muscle, NAD^+^, NADH, and NADPH increased in response to injury, but NAD^+^, NADH, NADP^+^, and NADPH were not affected by NRPT supplementation (Figure 3B). Pterostilbene sulfate was measurable in skeletal muscle after NRPT supplementation (Figure 3B).

### NRPT does not improve muscle function after injury.

**Figure 4:**
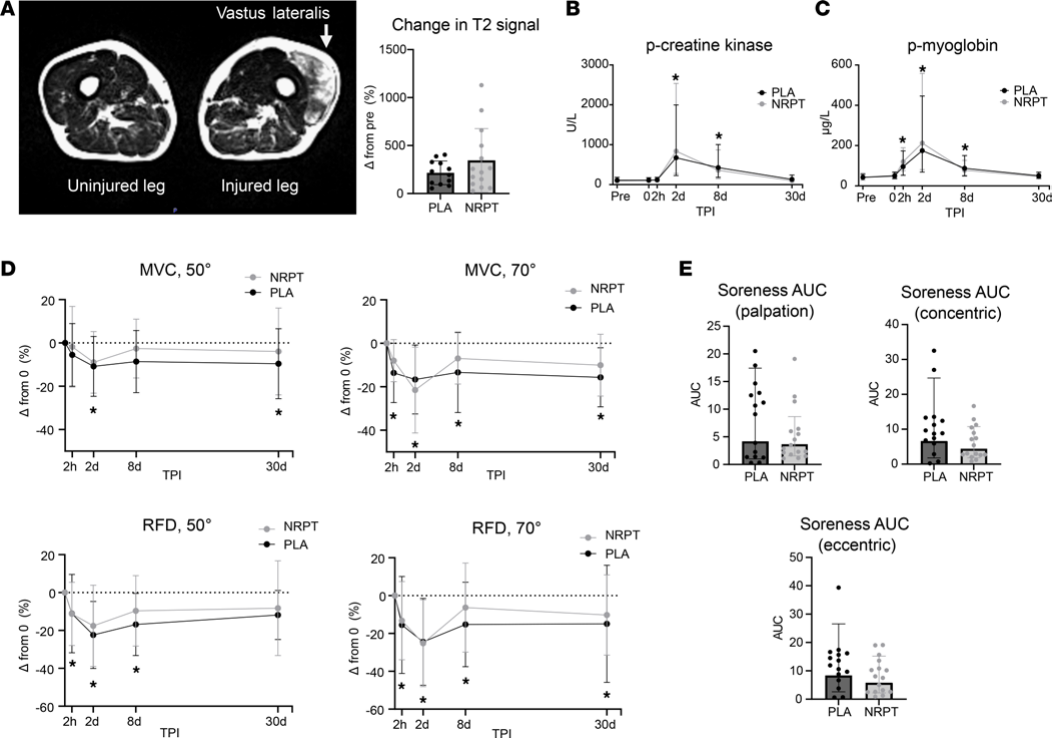
Clinical parameters associated with skeletal muscle injury. (**A**) T2-weighted axial image of the thigh shows increased signal intensity in the vastus lateralis part of the quadriceps femoris muscle 8 days after injury, with no difference between groups (NRPT, *n* = 14; PLA, *n* = 12). (**B**) P-creatinine kinase, (**C**) p-myoglobin, (**D**) maximal voluntary contraction and rate of force development at 50- and 70-angle degrees (MVC, 50°/70° and RFD, 50°/70°), and (**E**) muscle soreness did response to injury but not to NRPT supplementation (NRPT, *n* = 16; PLA, *n* = 15). Data are expressed as geometric mean ± SD, with the exception of MVC and RFD data in **D**, which are expressed as mean ± SD. Data were compared using repeated-measurement mixed-model analysis, with the exception of MRI and muscle soreness data, which were compared using a Student’s *t* test. *P < 0.05 vs. Pre. TPI, time after injury; MVC, maximal voluntary contraction; RFD, rate of force development; AUC, area under the curve.

The injury protocol induced significant clinical signs of muscle injury, such as muscle soreness, decrease in muscle strength, and release of muscle enzymes (Figure 4). T2 signal showed localized water infiltration in the vastus lateralis part of the quadriceps femoris muscle 8 days after injury (Figure 4A). No difference in signal intensity was detected between NRPT and placebo. A pronounced elevation of plasma (p-) myoglobin was observed already 2 hours after injury, and it remained elevated until 8 days after injury, whereas p-creatine kinase levels were only elevated 2 days and 8 days after injury (Figure 4, B and C). No supplementation effect was observed. Functional recovery, measured as maximal voluntary contractions (MVC) and rate of force development (RFD) of the quadriceps femoris muscle, was assessed at 2 knee angles (50 and 70 degrees). Both MVC and RFD were decreased after injury with no effect of the supplementation (Figure 4D). Accumulated muscle soreness indicated a tendency of lower soreness with NRPT supplementation (Figure 4E); however, this difference did not reach statistical significance.

### NRPT does not alter local cellular response to muscle injury.

**Figure 5:**
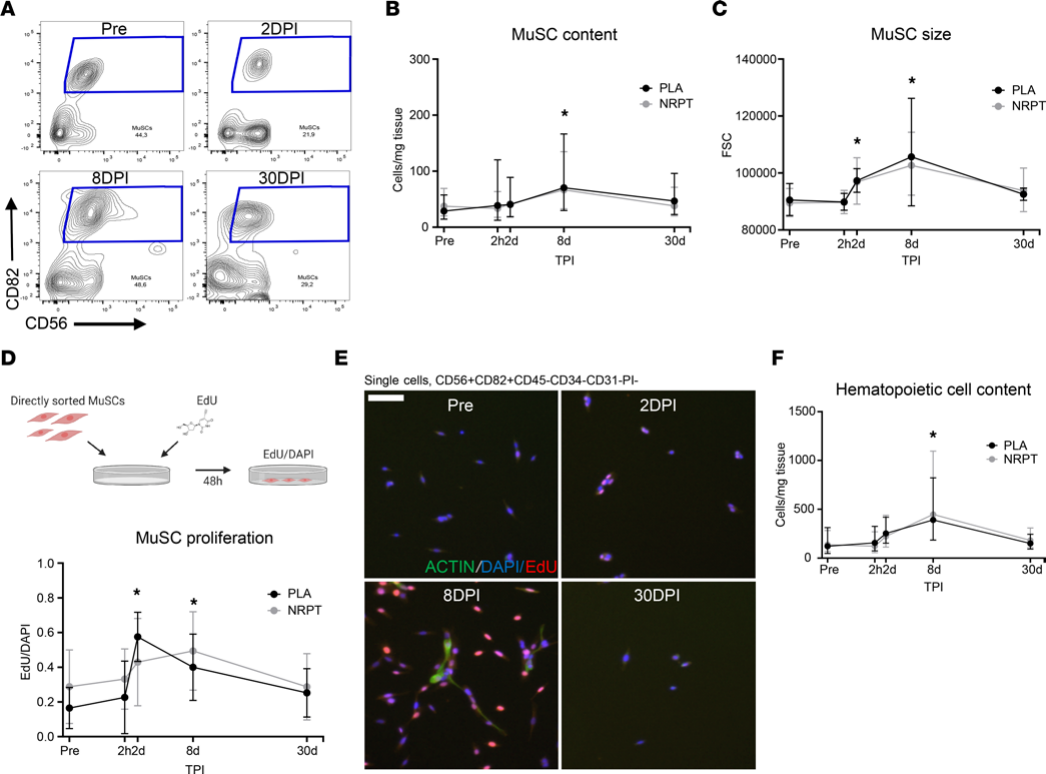
Muscle stem cell response to skeletal muscle injury. (**A**) CD56 (neural cell adhesion molecule 1) versus CD82 (KAI1) contour flow plots of single CD45^–^CD31^–^CD34^–^PI^–^ cells from skeletal muscle biopsies before, 2, 8, and 30 days after injury. (**B**) MuSCs content was quantified per milligram of skeletal muscle tissue, and (**C**) size was measured from forward scatter (FSC) of flow cytometry. (**D** and **E**) As a measure of MuSC proliferation, MuSCs were sorted and ex vivo EdU incorporation was measured after 48 hours of incubation and expressed relatively to DAPI. Scale bar: 75 μm. (**F**) Total content of hematopoietic cells (CD45^+^). Data are expressed as geometric mean ± SD, with the exception of MuSC proliferation data, which is expressed as mean ± SD, and compared using repeated-measurement mixed-model analysis. NRPT, *n* = 16; PLA, *n* = 15. *P < 0.05 vs. Pre. DPI, days after injury; MuSC, muscle stem cell; TPI, time after injury.

Flow cytometry and FACS were utilized for absolute quantification and sorting of MuSCs (CD56^+^CD82^+^CD45^–^CD34^–^CD31^–^ population) based on established protocols (22, 23). This cell population expresses high levels of PAX7 (a common MuSC marker) and can spontaneously undergo myogenic differentiation and myotube formation (22, 23). The absolute MuSC content increased by 107% from before injury to 8 days after injury, with no difference shown between NRPT and placebo (*P* = 0.58) (Figure 5, A and B). When stem cells activate from quiescence, going toward cell cycle entry, the cell size increases, which partly reflects anabolic processes. To increase resolution of initial MuSC activation, cell size was measured by forward scatter from flow cytometry. Two days after injury, mean cell size was increased by 8%, with a maximum increase of 18% observed 8 days after injury. No effect of NRPT supplementation was detected (Figure 5C). To examine initial cell-cycle entry of the MuSC pool, highly pure MuSCs were directly isolated, and ex vivo EdU incorporation was evaluated 48 hours after sort. Before injury, 23% of isolated MuSCs were positive for EdU; this was increased by 113% and 96% 2 days and 8 days after injury, respectively. This confirms a high proliferative rate of the MuSC pool at these 2 time points but with no effect of supplementation (Figure 5, D and E). However, we did note a tendency toward a significant supplementation × time interaction in MuSC EdU incorporation (*P* = 0.05).

The inflammatory response and its modulation is an essential part of the regeneration process and MuSC function; we therefore examined the content of hematopoietic cells (CD45^+^). CD45^+^ cell content was increased by 236% 8 days after injury, but no effect of NRPT supplementation was observed (Figure 5F). Overall, these data show pronounced effects of induced injury, but with no effect of NRPT on cellular response to muscle injury.

### NRPT does not affect histological changes in response to injury.

**Figure 6:**
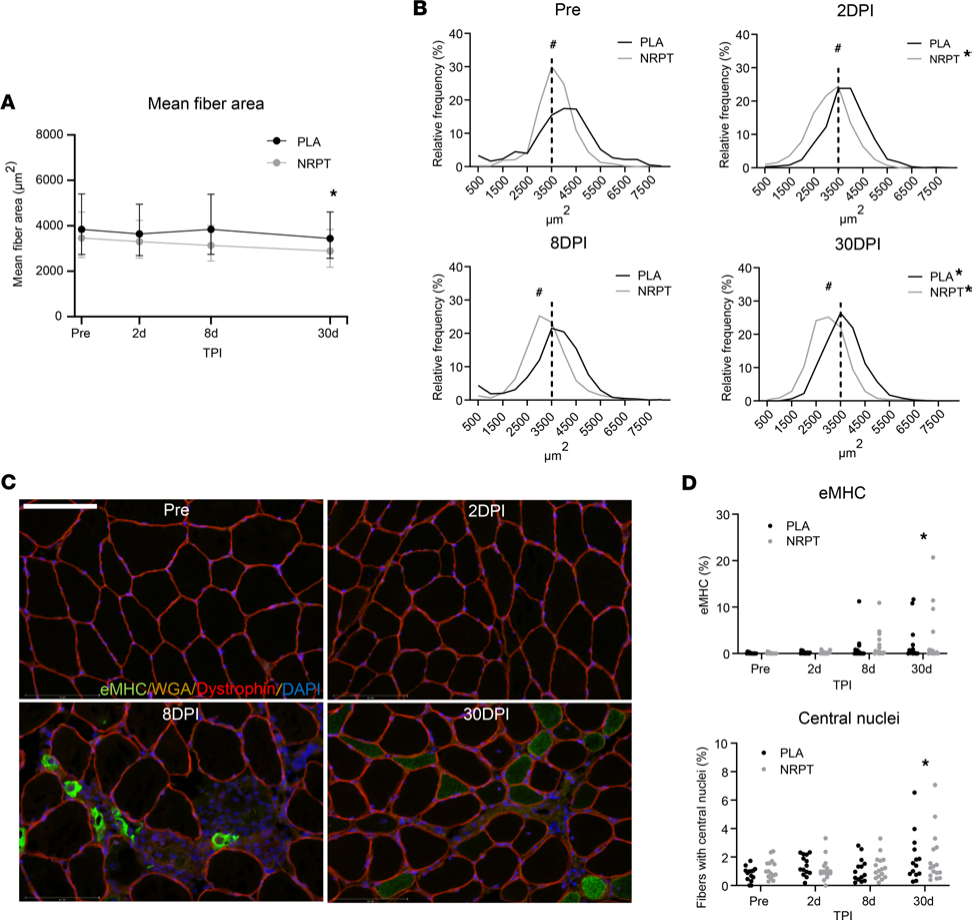
Histological analysis of myofibers. (**A**) Mean fiber area was decreased 30 days after injury. (**B**) This was supported by a left displacement of the fiber area distribution plot. (**C**) Immunostaining revealed areas with infiltrated muscle fibers and clearly revealed expression of embryonic myosin heavy chain (eMHC). Scale bar: 125 μm. (**D**) Both eMHC-expressing fibers and fibers with central nuclei (expressed per 100 muscle fibers) were increased 30 days after injury. Mean fiber area data are expressed as mean ± SD and compared using repeated-measurement mixed-model analysis. χ^2^ test was used to evaluate distribution of skeletal muscle fiber area. For eMHC and central nuclei data, each group was tested separately using Friedman’s test, and for supplementation effect, groups were compared at each time point after injury with the Mann-Whitney test. NRPT, *n* = 16; PLA, *n* = 15. ^#^*P* < 0.05 between groups; *P < 0.05 vs. Pre. TPI, time after injury; DPI, days after injury.

To study the effectiveness of the regenerative response, histological sections were examined for changes in muscle fiber area and markers of newly generated fibers (central nuclei and expression of eMHC). Mean fiber area was decreased 30 days after injury (Figure 6A). This was supported by left displacement of the fiber area distribution plot, indicating an increase in small and newly generated fibers after injury (Figure 6B). Muscle fiber area distribution was smaller in the NRPT group compared with the placebo group at all time points (including before injury). The number of fibers containing central nuclei and eMHC were increased 30 days after injury with no effect of NRPT supplementation (Figure 6, C and D).

## Discussion

The main finding of this study is that oral supplementation with NRPT did not improve recruitment of the MuSC pool after muscle injury in healthy elderly individuals. The MuSC content 8 days after injury was chosen as the primary endpoint in this trial. MuSC content is not a direct measurement of MuSC activation per se. However, the primary endpoint was supported by secondary outcomes, such as MuSC size and cell-cycle entry kinetics, and neither primary nor secondary endpoints supported a role of NRPT in treatment of muscle injury.

The injury protocol induced substantial skeletal muscle damage, as confirmed by muscle edema (T2 signal), release of muscle enzymes, reduced muscle function, and soreness. The quantities of muscle enzyme release in these elderly individuals were equivalent to those found after application of a similar protocol in elderly men (60–73 years) but inferior to those in younger men (24), which probably reflects a greater quantity of muscle mass affected in the young. We found decreased muscle strength of the quadriceps femoris muscle by approximately 25% 2 days after injury. This underlines the potency of the protocol because T2 signal 8 days after injury documented that only the vastus lateralis part of the quadriceps femoris muscle was directly injured. Furthermore, infiltration of immune cells and histological analysis of skeletal myofiber turnover confirmed a substantial myofiber injury and subsequent regeneration up to 30 days after induction of injury, which, to some extent, resembled that of experimental animal muscle injury protocols (25).

We found no effect of NRPT supplementation on either quantity or quality of MuSCs. In animals, both MuSC quality and quantity decline during aging (3), and these declines have been shown partly reversible (16, 17, 26). Human MuSC quantity also declines during aging (27, 28), but the effect on MuSC quality has not been investigated to the same degree. Recent evidence suggests that a major determinant of MuSC quality is the speed at which the cell can activate from quiescence to perform first cell division. Consequently, this speed is essential for injury recovery rate in mice (29). In humans, time to first cell division is shown to be approximately 83 hours for MuSCs (from the latissimus dorsi muscle) from an uninjured muscle (30). To examine MuSC transition and cell-cycle entry kinetics, we performed EdU incorporation during the first 48 hours after isolation. This revealed a marked increase in cycling MuSCs at 2 days after injury, although the total content of MuSCs was not yet increased. This was consistent with an increase in MuSC size 2 days after injury, suggesting that the MuSCs were indeed activated and undergoing an anabolic process in response to injury, which is similar to animal reports (29, 31). The increase in MuSC proliferation was maintained 8 days after injury, while the MuSC size further increased. The latter is likely reflecting a larger pool of cycling MuSCs with a high metabolic and cellular turnover to support further cell divisions. Thus, 2 days after injury, MuSC size more readily represented cell-cycle entry and cycling, whereas 8 days and 30 days after injury, MuSC size might also reflect exit and committed myogenic differentiation. These data provide potentially novel insight into the cell-cycle kinetics of human MuSCs in response to injury and suggest a high degree of similarity to data from animal models. Moreover, this brings the possibly novel approach of characterizing mononuclear cells in skeletal muscle biopsies from human interventional studies.

Oral intake of NR can increase NAD^+^ levels in circulating leucocytes as well as affect the skeletal muscle NAD^+^-metabolome in humans (21, 32–35). We found similar increases in NAD^+^ in whole blood after NRPT supplementation, but this did not translate into increased uptake in skeletal muscle tissue. This illustrates that whole blood cannot be used to determine muscle exposure to NR in clinical trials. Interestingly, NAD^+^ levels increased in the muscle biopsy material in response to injury. This is most likely a reflection of the substantial cellular displacement and infiltration of immune cells in muscle tissue 8 days after injury. Recent human trials have shown no effect of oral intake of NR on skeletal muscle mitochondrial content, structure, and bioenergetics (33–36) which contrasts findings in animals (17). This could be due to lower NR doses in human trials or because NR has been used in people with preserved NAD^+^ levels. However, proof of concept for improved muscle strength and mitochondrial biogenesis has been determined using nicotinic acid (NA), an alternative NAD^+^-precursor, in patients with Adult-Onset Mitochondrial Myopathy (37). These patients had both lower systemic and muscle NAD^+^ levels compared with healthy controls, which were rescued following NA supplementation. Therefore, it is important not to preclude potential benefits of NRPT supplementation in populations suffering from low NAD^+^ levels.

Despite the randomized design, the mean age differed significantly between the placebo group (63 years) and NRPT group (69 years). Therefore, a type-two-error of no supplementation effect cannot be excluded. However, we observed no correlation between age and MuSC response to injury, which could have explained the results (Supplemental Figure 1). In addition, MuSC content, size, and proliferation did not correlate with age in preinjury biopsies (Supplemental Figure 1). Finally, skeletal muscle fiber size is known to decrease during aging, and we found a similar trend in the preinjury biopsies (data not shown). Thus, we cannot exclude that the greater frequency of small myofibers in the NRPT group is related to the difference in age. Overall, our findings of no effect of NRPT supplementation on recruitment of the MuSC pool do not seem to be driven by a difference in age.

While our study was not optimized to determine effects of NRPT on all secondary endpoints, we did notice a tendency toward lower muscle soreness in NRPT group. Potential positive NRPT effects on muscle ache could be speculated to influence daily activity. However, physical activity and performance in muscle strength tests did not correlate with muscle soreness, so indirect effects mediated by reduced muscle pain are unlikely. In this study, we chose to determine the combined effect of NRPT on muscle regeneration in elderly individuals. The 2 previous human studies showing effects of NRPT on muscle strength used a duration of 2–4 months of supplementation (20, 21). An effect of longer duration of preinjury supplementation, the individual components, or NRPT effects on skeletal muscle metabolism in uninjured conditions cannot be ruled out.

In conclusion, supplementation with NRPT (1,000 mg NR and 200 mg PT) daily is safe but does not improve recruitment of the MuSC pool and, thereby, skeletal muscle regeneration after substantial injury in elderly human individuals.

## Methods

### Study population.

Thirty-two men and women were recruited to the study through local media. Inclusion criteria were met, if participants were aged 55–80 years (women should be postmenopausal), were nonsmokers, had a BMI of 20–28 kg/m^2^, had less than 2 hours of physical activity per week, and provided written informed consent. Exclusion criteria included endocrine disease, neurological or muscle disease, other severe disease, or more than 30 minutes of physical activity per day. Before enrollment, participants underwent a physical examination including routine clinical biochemistry and electrocardiography (Table 1). Three participants were treated for mild hypertension and received 50 mg losartan (*n* = 2) and 2.5 mg bendroflumethiazide and 573 mg potassium chloride (*n* = 1). Four participants were using D vitamin and calcium, 3 were using magnesium and zinc as a supplement. Participants were asked to refrain from supplementation with vitamins and dietary supplements 2 weeks before enrollment. Furthermore, participants were asked not to make dietary changes during the study period.

### Study design.

The study was an investigator-initiated randomized, double-blinded, placebo-controlled trial. Participants received oral supplementation with NRPT (NR, 500 mg; PT, 100 mg) (Basis, Elysium Health) twice daily or matched placebo. The pharmacy at Aarhus University Hospital was responsible for randomization, blinding, packaging, and labeling. Block randomization was used with a block size of 4: 2 receiving placebo and 2 receiving NRPT in a random pattern. Participants and investigators were blinded to supplementation. Once all participants had completed the study, and data were analyzed, the randomization code was released. For safety evaluation participants were systematically interviewed for potential adverse events by a trained physician 14 days after initiation of supplementation as well as at the end of the study period, and routine blood biochemistry was obtained 14 days after initiation of supplementation.

### Muscle injury protocol.

Participants were randomly assigned to injury in their dominant or nondominant leg. Skeletal muscle injury was induced by adapted protocol (38). Participants were seated upright in a dynamometer (HumacNorm 1, CSMI Inc.), and the chair position was adjusted so the center of the knee was aligned with the turning point of the dynamometer. The length of the arm was set 3 cm above the lateral malleolus. Stimulation patches (5 × 10 cm) were placed over the vastus lateralis muscle approximately 10 cm below anterior superior iliac spine and 5 cm above patella. The stimulator (Elpha II/3000, Biofina) delivered a current at a frequency of 35 Hz, 300 μs pulse duration, 0–100 mA, with an increasing phase of 0.5 seconds, plateau of 5 seconds, and decreasing phase of 0.5 seconds every fifth second. Applied mA was controlled by the participant, and they were regularly encouraged to go to highest tolerable level. When stimulation caused extension in the knee joint to 20 degrees, the lower leg was automatically pushed in the opposite direction by the dynamometer arm with a range of motion of 90 to 20 degrees (0 degrees = full extension). Five sets of 20 contractions were completed at 30 degrees/s and 180 degrees/s, respectively. Participants had a familiarization phase of 5–10 contractions without eccentric load before initiation. Moment-angle curves were recorded, expressed relatively to body weight, and total work calculated as sum of integrated curves using angles expressed in radians.

### Muscle strength test.

MVCs and RFD of extension in the knee joint were measured in both legs in the dynamometer at 70- and 50-degree joint angles. Three contractions were measured at each angle (duration = 2–4 seconds, with 30 seconds rest in between). Muscle strength test was performed approximately 20 minutes after muscle biopsy at all time points except from day 0 (measured immediately after injury). MVC was determined as the highest measured value. RFD was determined as the initial slope of the moment-time curve from 0 to 100 ms.

### Muscle soreness.

Muscle soreness was assessed in both legs on a continuous visual analog scale ranging from 0 to 10 cm (0 = no pain and 10 = worst thinkable pain) by self-palpation of the vastus lateralis part of the quadriceps femoris muscle using 3 fingers (palpation), active flexion with no load (flexion), and squad (eccentric). Soreness was assessed first thing in the morning before injury and on 0 days (immediately before and after injury protocol), 1 day, 2 days, 3 days, 4 days, 5 days, 6 days, and 7 days after injury. Participants received supervision the first 3 times. Area under the curve of soreness score (*y* axis) and time (*x* axis) was compared between groups.

### Blood analysis.

Blood analysis of creatinine kinase and myoglobin was carried out at the Department of Clinical Biochemistry, Aarhus University Hospital. Whole blood for NAD^+^ metabolites and pterostilbene sulfate analysis were collected in EDTA tubes and placed on ice until storage at –80°C.

### UHPLC-MS analysis of whole blood.

Method was adapted and developed according to Lu et al. (39). 100 μL whole blood was mixed with 20 μL isotopically labeled internal standard mix and 380 μL extraction solvent (methanol/water = 8:2; V/V). Internal standard mix included 10 mg/L nicotinic-d4 acid, nicotinamide-d4, N-methylnicotinamide-d4, and D-tryptophan-d5 (all from CDN Isotopes); adenosine-^13^C_5_ and nicotinamide adenine dinucleotide-d4 (Toronto Research Chemicals); and adenosine-^13^C_10_,^15^N_5_ 5′-triphosphate (MilliporeSigma). All internal standards were dissolved in methanol and were used to control extraction efficiency and method variation. Extraction was made using an ultrasound ice-cold bath for 10 minutes. Proteins were removed overnight at –20°C. Before centrifugation (11,292*g*, 5 minutes, 4°C), samples were reequilibrated at room temperature. After centrifugation, 180 μL supernatant was taken and evaporated to dryness under a stream of nitrogen and reconstituted with 50 μL methanol/water (8:2; V/V). Calibration curves were prepared in the same way by replacing sample with metabolite standards.

NAD^+^ metabolites and pterostilbene sulphate were analyzed using ultra-high performance liquid chromatography with mass spectrometry detection. Liquid chromatograph (Agilent, 1290 Infinity II) was coupled with mass spectrometer (timsTOF Pro, Bruker Daltonics). Metabolite separation was achieved using a 1.7 μm, 2.1 × 150 mm Waters ACQUITY Premier BEH Amide column. Mobile phase A was miliQ ultrapure water, including 10 mM ammonium acetate and 5 μM medronic acid. Mobile phase B was composed of acetonitrile and water (V/V = 9:1), again with the same amount of ammonium acetate and medronic acid. Gradient started with 90% of mobile phase B where it was stable for 2 minutes. Thereafter, it was decreased to 55% in 10 minutes where it was held for 2 minutes. At the end, the initial composition of mobile phases was restored. Injection volume was 2 μL. Mass detector was set to analyze exact mass spectra in full-scan mode in the range from 50 to 1,000 Da, at 1 Hz. Mass resolution was more than 60,000. Metabolites were identified by exact mass and retention time of metabolite standards with exception of pterostilbene sulfate, which was confirmed using fragmentation spectra. Quantification was made with dilution series of metabolite standards. Concentration was expressed in μM. Pterostilbene sulfate abundance was assigned with method specific arbitrary unit.

### Dual-energy X-ray absorptiometry.

Body composition were assessed by a whole-body DXA scan at Department of Endocrinology, Aarhus University Hospital (Hologic Discovery, Hologic). Two participants in the NRPT group did not undergo the second scan.

### MR imaging.

MR imaging was carried out before and 8 days after injury on a Siemens Skyra 3T-MR-scanner. Participants were placed supine, and an 18 element anterior coil was positioned over both thighs. Localizer images were acquired to define the length of the vastus lateralis muscle. T2 maps were acquired by a multiecho spin echo sequence with a field of view of 420 × 289 mm, matrix of 256 × 176, and 8 slices with thickness of 7 mm. Repetition time was 2.7 seconds and 16 echoes, and echo times from 20 ms to 320 ms were acquired. Scan time was 3 minutes. T2 maps were obtained by fitting an exponential function to the images. T2 signal was quantified in PMOD version4.0 (PMOD Technologies Ltd). A volume of interest was generated as 3–4 axial images in the middle of the vastus lateralis muscle, where the muscle is well-defined. Due to technical issues, data from only 26 individuals were obtained.

### Activity level and energy expenditure.

Participants wore a combined HR monitor and accelerometer (Actiheart4, CamNtech Ltd.) horizontally attached over the left third intercostal space using electrocardiogram electrodes (SP-50, Pulse Medical). The Actiheart was initialized using 60-second epochs and synchronized with a digital clock before mounting. The software provides an estimate of energy expenditure using branched modeling considering both HR and activity measurements with input of resting HR, weight, and age. Data on activity (counts/minute), mean HR (beats/minute), resting energy expenditure, activity energy expenditure, and total energy expenditure (mean Kcal/day) were extracted from the Actiheart software. The device was worn from 2 days before injury to 8 days after injury. Days with more than 120 lost epochs were excluded from analysis. Data collection was lost in 1 participant in the placebo group and 1 participant in the NRPT group. Overall, an average of 7.9 days per participant were included.

### Muscle biopsy.

Muscle biopsies were obtained from the vastus lateralis muscle with a Bergström needle with manual suction under local analgesics (10 mg/mL Xylocaine, AstraZeneca) in sterile conditions. Preinjury biopsy was collected from the noninjured leg, and 4 biopsies were collected from the injured leg (2 hours, 2 days, 8 days, and 30 days after injury). To minimize the effect of repeated sampling, a minimum distance of 3 cm between incisions was ensured. In addition, participants were stratified into 3 different biopsy location patterns. Biopsy material for histology was embedded in Tissue-Tek (Sakura Finetek Europe), frozen in precooled isopentane, and stored at –80°C. Biopsy material for FACS was weighted and stored in C tubes (130-093-237, Miltenyi Biotec) containing 6.5 mL ice-cold wash buffer (Hams F10 incl. glutamine and bicarbonate [N6908, MilliporeSigma]; 10% horse serum [26050088, Gibco, ThermoFisher Scientific], 1% Penicillin-Streptomycin [15140122, Gibco]) for a maximum of 2 hours before digestion.

### Tissue digestion.

Prior to digestion of muscle tissue, Collagenase II (46D16552, Worthington) and Dispase II (04942078001, Roche Diagnostics) were added to the C tube, making a final concentration of 700 U/mL and 3.27 U/mL, respectively. Mechanical and enzymatic muscle digestion was then performed on gentleMACS (130-096-427, Miltenyi Biotec) for 60 minutes using a skeletal muscle digestion program. The solution was added to 10 mL wash buffer, filtered through a 70 μm cell strainer, and washed twice to collect any remaining cells, before centrifugation at 500*g* for 5 minutes. The supernatant was removed, and the cell pellet was resuspended in freezing buffer (130-109-558, StemMACS, Miltenyi Biotec) and stored at –80°C.

### Flow cytometry and cell isolation.

The protocol and antibody panel for flow cytometry and cell isolation have been validated and described in detail elsewhere (23). Cells were sorted into 5 mL collection tubes containing 500 μL wash buffer at 4°C.

### Cell proliferation.

MuSC proliferation was detected using a 5-ethynyl-2′-deoxyuridine (EdU)/click-it assay (C10337 and C10340, Invitrogen). Experiments were carried out in 96-well half-area plates (Corning) coated with ECM (E1270, MilliporeSigma). MuSCs were plated in wash buffer and 10 μM EdU immediately after sorting. After 48 hours, cells were fixed in 4% paraformaldehyde for 8 minutes, washed 3 times for 5 minutes each time in PBS, and kept in PBS at 4°C. To detect EdU incorporation, we followed the manufacturer’s protocol and counterstained with DAPI (1:50.000, D3571, Invitrogen, Thermo Fisher Scientific). Images were automatically acquired using an EVOS M7000 automated imaging system (Thermo Fisher Scientific) to ensure similar treatment of all wells. EdU- and DAPI-positive cells were semiautomatically counted in ImageJ (NIH) and expressed relative to each other.

### Histology and immunohistochemistry.

Cryosections from before and 2 days, 8 days, and 30 days after injury were used for histology (thickness of 10 μm). After thawing to room temperature, sections were fixed for 5 minutes in Histofix (01000, Histolab Products AB) and blocked using 1% BSA plus 10% FBS and 0.5% Triton in 1× PBS for 60 minutes. Primary antibodies targeting eMHC (mouse, 1:8, F1.652, Developmental Studies Hybridoma Bank) and Dystrophin (rabbit, 1:500, ab15277, Abcam) in 1% BSA plus 10% FBS in 1× PBS were added and incubated overnight at 4°C. Sections were then washed twice for 5 minutes each time in PBS and incubated with secondary antibody Alexa Fluor 488 goat anti-mouse and Alexa Fluor 647 goat anti-rabbit (1:500, A-11001, A-21245, Thermo Fisher Scientific) plus WGA Texas Red-X Conjugate (1:500, W21405, Thermo Fisher Scientific) for 60 minutes at room temperature. Finally, sections were washed in PBS 3 times for 5 minutes each time, with 1 wash containing DAPI (1:50.000, D3571, Invitrogen, Thermo Fisher Scientific), and mounted with cover slides. Minus primary controls were included for optimization to ensure specificity.

### Imaging and quantification.

Images were acquired using an EVOS M7000 automated imaging system. For eMHC, central nuclei, and fiber area quantification, whole biopsy sections were scanned and stitched to 1 image. Areas containing length cut fibers were removed from analysis. eMHC and central nuclei were manually counted in ImageJ (NIH) and expressed relative to total counted myofibers. An average of 527 ± 188 fibers were analyzed per biopsy. For fiber area quantification, semiautomatic muscle analysis using segmentation of histology (40) was used on dystrophin-stained sections. An average of 389 ± 126 fibers were analyzed per biopsy.

### UHPLC-MS analysis of muscle tissue.

The method used was previously reported by Damgaard et al. (41), and it was modified to fit the specific matrix and sample size. Briefly, muscle biopsies were freeze dried and pulverized under cryogenic conditions using dry pulverizer (CPO2, Covaris). Powder was weighed and amount of tissue (mg) was used for normalization of chromatographic peak areas.

Each sample of powdered tissue was mixed with 200 μL methanol, which contained 0.02 mg/L of 3-hydroxy tridecanoic acid (IS, Cayman). Suspension was vortexed, and extraction was assisted using an ultrasound bath for 10 minutes at room temperature. Suspension was left on ice in order to precipitate proteins for 20 minutes. After precipitation, samples were centrifuged on 11,292*g* at 4°C for 3 minutes. 120 μL of extract was transferred into a HPLC vial, and 20 μL was used to prepare a pooled sample which was used for quality control purposes. Extracts were evaporated to dryness under a stream of nitrogen and were resuspended with 60 μL of methanol and water (V/V = 1:1).

Equipment used was the same as for whole blood analysis. Separation was based on reversed-phase chromatography. A Waters TSS T3 10 cm × 2.1 × 1.8 μm column was used. Mobile phase composition was as follows: mobile phase A was water, and mobile phase B was a mixture of acetonitrile and isopropanol (V/V = 3:1). Both mobile phases contained 0.1% formic acid. Chromatographic run started with 3% of mobile phase B and was gradually increased up to 100% over 9 minutes, where it was held stable for an additional 5 minutes. After that, the system was reequilibrated to initial conditions. The flow rate was 400 μL/min. Column temperature was kept at 40°C, and injection volume was 4 μL. To maximize the sensitivity of the mass spectrometer at relatively low-molecular masses, a screening method was developed that acquired spectra between 0 and 500 Da. Scan speed was set to 2 Hz, and acquisition was performed in negative ionization mode.

Row data were converted to MzML format, and MzMine 2.53 (42) was used for targeted peak area extraction. Pterostilbene sulfate chromatogram areas were normalized against internal standard abundance and against mass of the tissue in each sample. Normalized peak areas were assigned with method specific arbitrary unit.

### Analysis of NAD^+^ metabolites in muscle tissue.

For NAD^+^ and NADP^+^ analysis, freeze-dried muscle samples were lysed in 400 μL 0.6 M perchloric acid (HClO_4_) using steel beads and a TissueLyser II. Samples were subsequently centrifuged for 2 minutes at 13,000*g*, and the supernatant was diluted 200 times in 100 mM Na_2_HPO_4_ (pH = 8). The remaining pellet was dissolved by heating to 95°C in 400 μL 0.1 M NaoH, and the protein level was measured (Pierce BCA Protein Assay Kit). For NADH and NADPH analysis, the samples were treated similarly, except they were lysed in 400 μL 0.1 M NaoH and heated for 10 minutes at 70°C before centrifugation, before a sample of the supernatant was taken for protein measurement prior to dilution (200 times) in 10 mM Tris HCl (pH = 6).

A total of 100 μL of each diluted sample was mixed 1:1 with appropriate reaction mix, and fluorescence levels were measured every minute for 30 minutes (excitation 544 nm, emission 580 nm). The slopes of the reactions were compared with standard curves and normalized to protein amounts.

The reaction mix for NAD^+^ and NADH was 5,000 μL 200 mM Na_2_HPO_4_, 3,500 μL milli-Q H_2_O, 1,200 μL 750 U/mL alcohol dehydrogenase, 200 μL absolute ethanol, 100 μL 1 M nicotinamide, 26 μL 50 U/mL diaphorase, 10 μL 10 mM flavin mononucleotide, and 5 μL 5 mg/mL resazurin.

The reaction mix for NADP^+^ and NADPH was 5,000 μL 200 mM Na_2_HPO_4_, 2,950 μL milli-Q H_2_O, 1,750 μL 200 mM glucose-6-phosphate, 150 μL 1,000 U/mL glucose-6-phosphate dehydrogenase, 100 μL 1 M nicotinamide, 26 μL 50 U/mL diaphorase, 25 μL 500 mM MgCl_2_, 10 μL 2 M NaoH, 10 μL 10 mM flavin mononucleotide, and 5 μL 5 mg/mL resazurin.

### Statistics.

Data are expressed as mean ± SD if normal distributed or geometric mean ± SD if not normal distributed. Data distribution was evaluated by inspection of QQ plots. A 2-tailed Student’s *t* test was performed to compare anthropometric, work, current, activity, soreness, and MRI data (log transformed). P-myoglobin (log transformed), p-creatinine kinase (log transformed), NAD^+^ metabolites, MVC data, MuSC data (log transformed), CD45^+^ data (log transformed), and mean fiber area (log transformed) were analyzed using a repeated-measurement mixed-model analysis with intervention, time, and the interaction between intervention and time as factors. In case of a significant interaction, pairwise comparisons were performed for differences within and between interventions and time and Dunnett’s method of multiple comparisons was performed. MuSC content in skeletal muscle biopsies 8 days after injury was used as primary endpoint for this trial. Assumptions for the power calculation were mainly based on a previous trial investigating the effect of ibuprofen on MuSC recruitment using a similar skeletal muscle injury protocol (43). In the study, MuSC content was quantified as PAX7-positive cells using immunostaining on cryosections and expressed relative to amount of myofibers. We expected a normal distribution of the endpoint after log transformation and a SD of 20% on the log scale. The minimal clinically relevant difference was set to 15% of the log-transformed estimate and the power to 80%. This equals to 16 individuals in each group. χ^2^ test was used to evaluate distribution of skeletal muscle fiber area. For central nuclei and eMHC, each group was tested separately with Friedman’s test and with Dunnett’s method for multiple comparisons if the outcome was significant. To test for a supplementation effect, groups were compared at each time point after injury with the Mann-Whitney test. Significance was set at *P* < 0.05. All analyses were carried out using GraphPad Prism version 8.3.0 and STATA version 16. Graphs were generated in GraphPad Prism, and figures were created with Biorender.com.

### Study approval.

The study was conducted in accordance with the Declaration of Helsinki and specific national laws after approval by the local research ethics committee in Central Denmark Region (1-10-72-301-18). The study was registered at Clinicaltrials.gov (NCT03754842) before recruitment was commenced. Participants received oral and written information before written consent was obtained.

## Author contributions

JBJ, JF, OLD, ABM, SR, NM, JTT, and NJ conceived and designed the experiments. JBJ, JF, TBB, SR, MVD, and KT performed the experiments. JBJ, JF, OLD, ABM, RWD, SR, ED, SC, KT, TM, MVD, JTT, and NJ analyzed and interpreted the data. JBJ and JF prepared the figures and drafted the manuscript. All authors read, revised, and approved the final manuscript. NJ is the guarantor of the work, has full access to all the data in the study, and takes responsibility for the integrity of the data and the accuracy of the data analysis.

## Supplementary Material

Supplemental data

ICMJE disclosure forms

## Figures and Tables

**Table 1:**
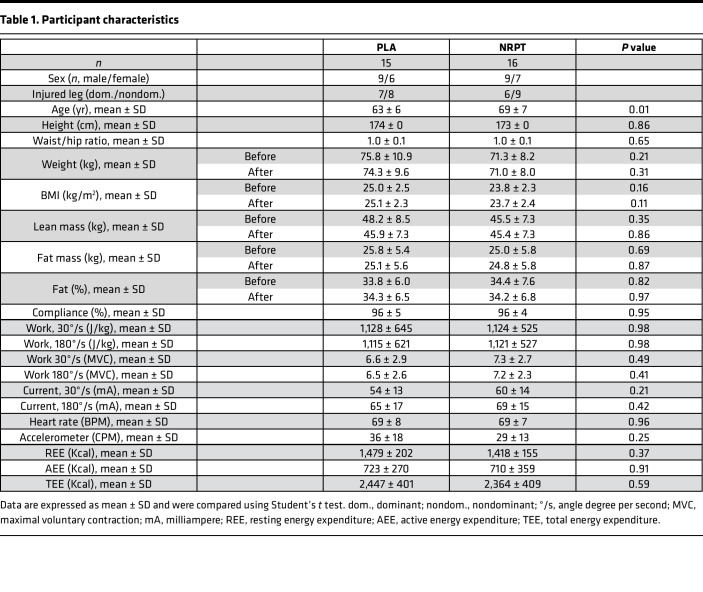
Participant characteristics

## Version 1 08/23/2022

In-Press Preview

## Version 2 10/10/2022

Electronic publication
