# Supplementary material for: A randomized placebo-controlled trial of nicotinamide riboside and pterostilbene supplementation in experimental muscle injury in elderly individuals
Source: JCI Insight. 2022 Oct 10;7(19):e158314. doi: 10.1172/jci.insight.158314 (PMC9675477; doi:10.1172/jci.insight.158314)
Supplement: Supplemental data [file jciinsight-7-158314-s196.pdf]

S1

		PLA	NRPT
**P-Alanintransaminase (U/L)**	**Pre**	28 ± 10	23 ± 8
	**Day 0**	29 ± 12	24 ± 11
**P-Potassium (mmol/L)**	**Pre**	4.1 ± 0.2	4.1 ± 1
	**Day 0**	4.1 ± 0.3	4.1 ± 0.2
**P-Sodium (mmol/L)**	**Pre**	140 ± 2	141 ± 2
	**Day 0**	140 ± 1	141 ± 2
**P-Albumin (g/L)**	**Pre**	41 ± 3	40 ± 2
	**Day 0**	40 ± 2	42 ± 3
**P-Creatinine (µmol/L)**	**Pre**	63 ± 11	66 ± 9
	**Day 0**	69 ± 13	70 ± 8
**P-Urea (mmol/L)**	**Pre**	5.8 ± 0.6	5.0 ± 1.2$^{\#}$
	**Day 0**	5.5 ± 0.9	4.8 ± 1.0$^{\#}$
**Leukocytes (10^9/L)**	**Pre**	5.3 ± 1.4	5.9 ± 1.6
	**Day 0**	5.5 ± 1.2	5.6 ± 1.9
**Hemoglobin (mmol/L)**	**Pre**	8.9 ± 0.5	8.5 ± 0.6
	**Day 0**	9.1 ± 0.7	8.9 ± 0.6
**Thrombocytes (10^9/L)**	**Pre**	240 ± 31	251 ± 69
	**Day 0**	252 ± 34	267 ± 84

S1: Blood biochemistry for safety evaluation.

Blood samples were obtained before enrollment (Pre) and 14 days after (Day 0) initiation of PLA or NRPT supplementation. P-Urea was significant different between groups at both Pre and Day 0. ($^{\#}$ = group effect).

S2

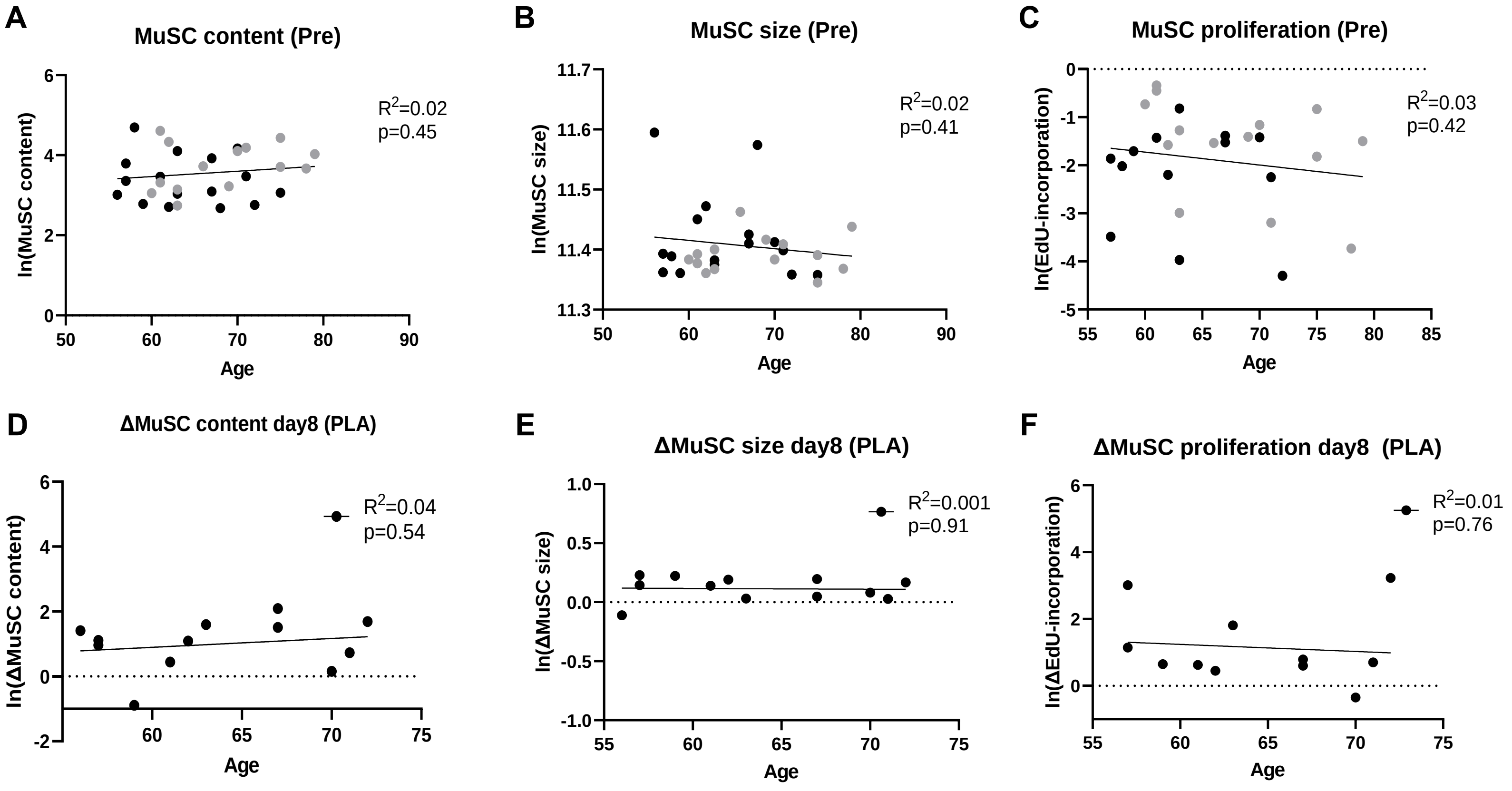

S2: Correlation between MuSC parameters and age

Correlation between age and MuSC content (A), size (B), and EdU-incorporation (C) from Pre biopsy. Correlation between age and change in MuSC content (D), size (E), and EdU-incorporation (F) from Pre to 8 days post injury in the placebo group.
